# Magnetic Resonance Imaging of Osteophytic, Chondral, and Subchondral Structures in a Surgically-Induced Osteoarthritis Rabbit Model

**DOI:** 10.1371/journal.pone.0113707

**Published:** 2014-12-01

**Authors:** Lang Jia, Jinyun Chen, Yan Wang, Yingjiang Liu, Yu Zhang, Wenzhi Chen

**Affiliations:** 1 State Key Laboratory of Ultrasound Engineering in Medicine Co-Founded by Chongqing and the Ministry of Science and Technology, College of Biomedical Engineering, Chongqing Medical University, Chongqing, China; 2 Department of Rehabilitation Medicine, the Second Affiliated Hospital, Chongqing Medical University, Chongqing, China; University of Massachusetts Medical, United States of America

## Abstract

**Objective:**

This study aimed to assess changes in osteophytic, chondral, and subchondral structures in a surgically-induced osteoarthritis (OA) rabbit model in order to correlate MRI findings with the macroscopic progress of OA and to define the timepoint for disease status in this OA model.

**Methods:**

The OA model was constructed by surgery in thirty rabbits with ten normal rabbits serving as controls (baseline). High-resolution three-dimensional MRI using a 1.5-T coil was performed at baseline, two, four, and eight weeks post-surgery. MRIs of cartilage lesions, subchondral bone lesions, and osteophyte formations were independently assessed by two blinded radiologists. Ten rabbits were sacrificed at baseline, two, four, and eight weeks post-surgery, and macroscopic evaluation was independently performed by two blinded orthopedic surgeons.

**Results:**

The signal intensities and morphologies of chondral and subchondral structures by MRI accurately reflected the degree of OA. Cartilage defects progressed from a grade of 0.05–0.15 to 1.15–1.30 to 1.90–1.97 to 3.00–3.35 at each successive time point, respectively (*p*<0.05). Subchondral bone lesions progressed from a grade of 0.00 to 0.78–0.90 to 1.27–1.58 to 1.95–2.23 at each successive time point, respectively (*p* = 0.000). Osteophytes progressed from a size (mm) of 0.00 to 0.87–1.06 to 1.24–1.87 to 2.21–3.21 at each successive time point, respectively (*p* = 0.000).

**Conclusions:**

Serial observations revealed that MRI can accurately detect the progression of cartilage lesions and subchondral bone edema over an eight-week period but may not be accurate in detecting osteophyte sizes. Week four post-surgery was considered the timepoint between OA-negative and OA-positive status in this OA model. The combination of this OA model with MRI evaluation should provide a promising tool for the pre-clinical evaluation of new disease-modifying osteoarthritis drugs.

## Introduction

Osteoarthritis (OA) is the most common joint disease affecting the elderly [1] and consists of a group of clinically heterogeneous disorders characterized by hyaline cartilage loss and subchondral bone reaction that cause debilating pain and a reduced ability to work [2]–[3]. As OA structural changes take place over decades in humans, it is understandably difficult to study the changes observed in the early stages of the disease. Thus, animal models that can reproduce the morphological and molecular changes in OA have been extensively used to study the pathophysiology of the disease [4], and some have been useful for testing drug therapies that have the potential to modify the evolution of OA (disease-modifying osteoarthritis drugs; DMOADs).

As the knee is one of the joints most commonly affected by OA [5], the surgically-induced OA model – which excises the medial collateral ligament, the medial meniscus, and both cruciate ligaments in the knee – has been shown to produce a slowly progressing OA in rabbits [6]. Moreover, three DMOADs applied in this surgically-induced OA model have displayed similar effects in the knees of OA patients [7]–[12]. Although there have been multiple studies reporting on OA development after knee destabilization with different endpoints and visualization methods [13]–[18], MRI findings have not yet been correlated with the macroscopic progress of OA in this surgically-induced OA rabbit model, and the timepoint of disease status in this OA model has not yet been defined.

In order to accomplish this task, selection of an appropriate imaging modality is paramount. Although radiological joint space narrowing by X-ray radiography is the “gold standard” for assessing OA, there is currently no well-established imaging modality to visualize changes in chondral and subchondral tissue in order to correlate these changes with more commonly utilized histolopathologic analysis and molecular biomarkers. To this end, the superior soft-tissue contrast and multiplanar capabilities of magnetic resonance imagaing (MRI) appear to make it the ideal technique for providing precise and reliable semi-quantitative information on changes in chondral and subchondral tissue structure [19], [20].

Therefore, in this study, MRI of a surgically-induced OA rabbit model was used to assess changes in osteophytic, chondral, and subchondral structures over a period of eight weeks in order to correlate these MRI findings with the macroscopic progress of OA. The severity of cartilage lesions, osteophytic growth, and subchondral bone edema were evaluated using semi-quantitative scoring systems in order to define the timepoint for disease status in this OA model. Establishing a method for MRI evaluation of this surgically-induced OA rabbit model should provide a promising tool for the evaluation of new DMOADs.

## Materials and Methods

### Ethics Statement

The experimental and animal care protocols were approved by the Committee on the Ethics of Animal Experiments at Chongqing Medical University (Chongqing, China). In addition, this study was performed in accordance with the recommendations described in the “Guide for the Care and Use of Laboratory Animals” from the Ministry of Science and Technology of the People's Republic of China. All surgery was performed under sodium pentobarbital anesthesia, and all efforts were made to minimize animal suffering during the course of this study.

### Rabbit Subjects & OA Model Construction

Forty male New Zealand White (NZW) rabbits (6–7 months, 2.0–2.5 kg) were obtained from the Animal Center at Chongqing Medical University. All animals were housed in individual cages with a 12:12-h light-dark cycle in 20–25°C and were given a standard laboratory diet and drinking water *ad libitum*.

In order to construct the OA model, the medial collateral ligament, the complete medial meniscus, and both cruciate ligaments were excised from both knees in 30 rabbits under general anaesthesia (3% pentobarbital, 1 ml/kg) as previously described [6]. The ten remaining normal rabbits served as controls. Activity, body weight, food consumption, rectal temperature, and wound healing were checked daily during postoperative week one. After postoperative week one, rabbits were induced to move in order to promote OA development for a half-hour daily over five days per week over a total period of seven weeks.

### MRI Procedure

Thirty model rabbits were randomly segregated into three experimental groups: the 2-week, 4-week, and 8-week groups (n = 10 rabbits per experimental group). All rabbits were anesthetized by 3% pentobarbital (1 ml/kg) prior to imaging, and both knees were examined simultaneously. MRI of both knees was performed with a l.5-T Flex Loop Small coil (Siemens) at 2, 4, and 8 weeks post surgery (depending on the experimental group), and the 10 control rabbits also had MRI performed at baseline. All examinations were standardised using a dedicated device allowing the rabbits to be placed in a supine position with the leg placed in the scanner at slight flexion.

We used a T2-FI3D-we-sag sequence (TR: 19 ms; TE: 9.5 ms; flip angle: 40^°^; slice thickness: 1 mm; field of view (FOV): 160 mm; FOV phase: 100%; scanning matrix: 512×512; voxel size: 0.6×0.6×1.0 mm; number of excitation (NEX): 1) for the cartilage and osteophyte analysis. A sagittal 2-D fast spin echo sequence (FSE) with fat saturation (TR: 3000 ms; TE: 98 ms; flip angle: 90^°^; slice thickness: 1 mm; FOV: 10 cm; matrix size: 384; NEX: 2) was used for the subchondral bone lesions as previously described [21]. The total acquisition time was approximately 12 min.

The images were prospectively analyzed by two blinded, independent musculoskeletal MRI radiologists. Radiologists were trained on the grading methods and were blinded to the macroscopic analysis results.

### Semi-Quantitative Scoring of Cartilage Defects

Cartilage defects were assessed in subregions of the knee [21], which included the anterior, posterior medial, and lateral femoral condyles; the anterior, central, posterior medial, and lateral tibial plateaus; and the trochlea. The cartilage defects were scored on a scale of grades 0 to 4 as described previously [22]: 0, normal cartilage; 1, abnormal intrachondral signal (either hypo- or hyperintense) with a normal chondral surface; 2, mild surface irregularity and/or focal loss of less than 50% of cartilage thickness; 3, severe surface irregularity with focal loss of more than 50% but less than 100% of cartilage thickness; and 4, complete loss of articular cartilage with exposure of subchondral bone. For each rabbit, the sum of the grades and the mean of the individual grades were calculated.

For the osteophytes, the medial and lateral tibial plateau were evaluated separately. The osteophytes were identified on the tibial plateau as local bone outgrowths on coronal MRI sections. The maximum width of osteophytic strips growing along the lateral and medial edges was measured, and the final score was the mean of the osteophyte size measured from both edges.

### Semi-Quantitative Scoring of Subchondral Bone Edema

The medial and lateral condyles and plateaus as well as trochlea were assessed. The lesion in each subregion was scored on a scale from 0 to 3 as previously described [19], [20]: 0, normal bone; 1, a hypersignal occupying less than 1/3 of the surface of the subregion; 2, a hypersignal occupying less than 2/3 of the surface of the subregion; and 3, a hypersignal occupying greater than 2/3 of the surface of the subregion. The lesion size in the subregions was assessed on the sections where the hypersignal was the greatest. The final scoring for each rabbit consisted of the sum of the grades and the mean of the individual grades for each subregion (i.e., femoral condyles and trochlea, plateaus, and compartments).

### Macroscopic Grading

Immediately after imaging, the 10 rabbits in each experimental group were sacrificed for macroscopic assessment of both knees. The macroscopic assessment was independently conducted by two blinded readers, both of which are trained and licensed orthopedic surgeons. The orthopedic surgeons performing the macroscopic analysis were trained on the grading methods and were blinded to the MRI results. Both knees were resected to examine the gross macroscopic morphologic changes as previously described [23]: 0, normal cartilage; 1, cartilage softening and/or swelling; 2, mild surface fibrillation and/or less than 50% loss of cartilage thickness; 3, severe surface fibrillation and/or loss of more than 50% of cartilage thickness but without exposure of subchondral bone; and 4, complete loss of cartilage with subchondral bone exposure. The maximal width (mm) of osteophytes on the medial and lateral tibial plateau were measured using a digital calliper (Digimatic Caliper, STARRETT, USA).

### Statistical Analysis

Statistical analysis was completed using SPSS v19.0 (IBM, USA). Measurement values were expressed as means ± standard deviations (SDs), and *p*<0.05 was considered significant for all analyses. We measured both knees and averaged the values from each knee into a single data point for our analyses. Prior to multiple comparisons, we tested whether the data was normally distributed and the variances were equal. If so, a one-way analysis of variance (ANOVA) with least significant difference (LSD) testing was used. If not, the non-parametric Kruskal-Wallis test was applied.

Weighted kappa statistics were calculated to assess the degree of interobserver agreement. The weightings were calculated with the formula 1- (|i - j|/[k - 1]), where i and j indicates the rows and columns of the ratings assigned by the two readers and k indicates the maximum number of possible ratings. A weighted kappa value of less than 0.00 indicates poor agreement, a value of 0.00 to 0.20 indicates slight agreement, a value of 0.21 to 0.40 indicates fair agreement, a value of 0.41 to 0.60 indicates moderate agreement, a value of 0.61 to 0.80 indicates substantial agreement, a value of 0.81 to less than 1.00 indicates almost perfect agreement, and a value of 1.00 indicates perfect agreement [24]. Pearson's correlation coefficient (r) was calculated to assess the degree of correlation between the MRI findings and macroscopic lesion grading across all time points.

## Results

### Rabbit Behavior and Activity

We monitored rabbit behavior and activity post-surgery by observing their response to pain, gait, and degree of joint swelling. We observed severe knee contraction in the affected knee (10/10 rabbits), severe joint swelling with disappearance of the knee's bony landmarks (10/10 rabbits), and walking incapacity (10/10 rabbits) were observed at week two post-surgery. Severe knee contraction in the affected knee (10/10 rabbits), severe joint swelling with disappearance of the knee's bony landmarks (8/10 rabbits), whole-body trembling (10/10 rabbits), and lameness (10/10 rabbits) were observed at week four post-surgery. Severe knee contraction in the affected knee (10/10 rabbits), lameness (10/10 rabbits), and joint deformity without joint swelling (10/10 rabbits) were found at week eight post-surgery.

### Cartilaginous Defects

**Figure 1:**
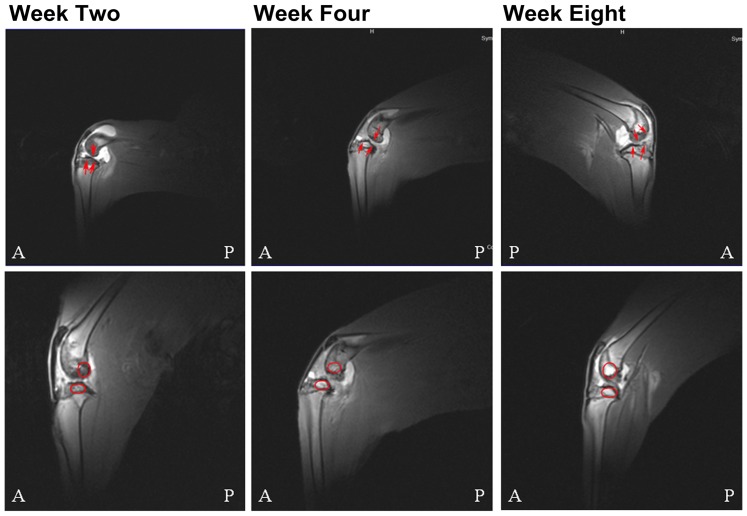
Representative Magnetic Resonance Images (MRI) of Rabbit Knees from Weeks Two through Eight Post-Surgery. Upper panels: Representative T2-FI3D-we-sag sequence MRI showing cartilage defects on the medial femoral condyles and tibial plateaus. Arrows indicate cartilage thickening (grade one) at week two post-surgery, cartilage edema or thickening with an intact surface (grades one to two) at week four post-surgery, and loss of cartilage (grades three to four) at week eight post-surgery. Lower panels: Representative sagittal spoiled gradient sequence MRI of femoral condyles, trochlea, and tibial plateaus showing the evolution of subchondral bone hypersignal from weeks two through eight post-surgery. A, anterior; P, posterior; circles indicate lesion areas (mm).

MRI of cartilagenous tissue showed that defects were found as early as week two post-surgery. At that time, cartilagenous lesions were more commonly observed on the medial condyles and plateaus (Fig. 1). Both MRI and macroscopic grading showed that the scores and grades of cartilagenous defects progressively increased over time (*p*<0.05). By week eight, cartilagenous lesions were present on all subregions (including the lateral region) but remained higher in the medial compartment (Table 1, 2).

**Table 1:** Cartilage Lesion Grades and Scores Measured by Magnetic Resonance Imaging (MRI).

		Femoral condyles and trochlea	Tibial plateaus	Medial compartment	Lateral compartment
**Baseline**	Score	2.00±1.00	1.50±0.71	2.50±0.71	1.50±0.71
	Grade	0.10±0.30	0.15±0.43	0.10±0.30	0.05±0.22
**Week two**	Score	24.33±2.08	25.50±2.12	26.00±1.41	23.00±1.41
		(20:20:20)	(20:20:20)	(20:20:20)	(20:20:20)
	Grade	1.22±0.42	1.27±0.45	1.30±0.46	1.15±0.36
**Week four**	Score	38.00±4.36*	39.23±2.83▴	39.00±1.41▾	33.50±0.71†
		(20:20:20)	(20:20:20)	(20:20:20)	(20:20:20)
	Grade	1.90±0.71•	1.94±0.56 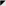	1.97±0.46Ф	1.90±0.55‡
**Week eight**	Score	64.33±3.06§	67.50±2.12¶ ^★^	65.00±2.83▪	62.50±3.54 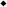
		(20:20:20)	(20:20:20)	(20:20:20)	(20:20:20)
	Grade	3.12±0.74^#^	3.35±0.75 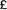	3.25±0.78 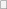	3.00±0.82 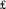

Data (n = 20 knees) are expressed as means±SDs. Scores are expressed as the summation of grades, and the grades as an average of the grades obtained for each region. Incidences of the lesions in subregions are indicated in parentheses (number of rabbits with lesions on medial/lateral femoral condyles/trochlea, medial/lateral tibial plateaus, or femoral condyles/tibial plateaus in the compartments).

Femoral condyles and trochlea score: week eight compared to weeks four and two,

§ *p* = 0.000; week four compared to week two,

^*^ *p* = 0.02. Femoral condyles and trochlea grade: week eight compared to weeks four and two,^ #^ *p* = 0.000; week four compared to week two,

• *p* = 0.000.

Tibial plateaus score: week eight compared to week four,

¶ *p* = 0.002; week four compared to week two,

▴ *p* = 0.022; week eight compared to week two,

★ *p* = 0.001. Tibial plateaus grade: Week eight compared to weeks four and two,

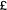
*p* = 0.000;week four compared to week two,

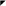
*p* = 0.000.

Medial compartment score: week eight compared to weeks four and two,

▪ *p* = 0.000; week four compared to week two,

▾ *p* = 0.022. Medial compartment grade: week eight compared to weeks four and two,

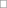
*p* = 0.000; week four compared to week two,

^Φ^ *p* = 0.022.

Lateral compartment score: week eight compared to weeks four and two,

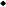
*p* = 0.000; week four compared to week two,

† *p* = 0.022. Lateral compartment grade: week eight compared to weeks four and two,

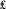
*p* = 0.000; week four compared to week two,

‡ *p* = 0.022.

**Table 2:** Cartilage Lesion Grades and Scores Measured by Macroscopic Examination.

		Femoral condyles nd trochlea	Tibial plateaus	Medial ompartment	Lateral ompartment
**Baseline**	Score	2.67±0.58	2.60±0.71	3.50±0.71	2.50±0.71
	Grade	0.12±0.32	0.20±0.41	0.18±0.38	0.08±0.27
**Week two**	Score	27.67±2.52	25.55±2.52	29.00±1.41	26.00±1.41
		(20:20:20)	(20:20:20)	(20:20:20)	(20:20:20)
	Grade	1.35±0.48	1.42±0.50	1.40±0.50	1.30±0.46
**Week four**	Score	38.67±1.53*	39.50±3.54▴	41.00±1.41▾	37.50±0.71†
		(20:20:20)	(20:20:20)	(20:20:20)	(20:20:20)
	Grade	1.93±0.66•	1.98±0.53 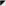	2.00±0.66Ф	1.88±0.52‡
**Week eight**	Score	68.00±1.73§	69.00±1.41¶ ^★^	69.50±0.71▪	67.50±0.71 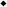
		(20:20:20)	(20:20:20)	(20:20:20)	(20:20:20)
	Grade	3.47±0.57#	3.45±0.5 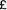	3.55±0.55 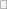	3.38±0.49 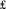

Data (n = 20 knees) are expressed as means±SDs. Scores are expressed as the summation of grades, and the grades as an average of the grades obtained for each region. Incidences of the lesions in subregions are indicated in parentheses (number of rabbits with lesions on medial/lateral femoral condyles/trochlea, medial/lateral tibial plateaus, or femoral condyles/tibial plateaus in the compartments).

Femoral condyles and trochlea score: week eight compared to weeks four and two,

§ *p* = 0.000; week four compared to week two,

^*^ *p* = 0.000. Femoral condyles and trochlea grade: week eight compared to weeks four and two,

# *p* = 0.000; week four compared to week two,

• *p* = 0.000.

Tibial plateaus score: week eight compared to week four,

¶ *p* = 0.001; week four compared to week two,

▴ *p* = 0.022; week eight compared to week two,

★ *p* = 0.001. Tibial plateaus grade: week eight compared to weeks four and two,

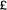
*p* = 0.000; week four compared to week two,

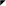
*p* = 0.000.

Medial compartment score: week eight compared to weeks four and two,

▪ *p* = 0.000; week four compared to week two,

▾ *p* = 0.022. Medial compartment grade: week eight compared to weeks four and two,

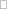
*p* = 0.000; week four compared to week two,

^Φ^ *p* = 0.000.

Lateral compartment score: week eight compared to weeks four and two,

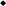
*p* = 0.000; week four compared to week two,

† *p* = 0.022. Lateral compartment grade: week eight compared to weeks four and two,

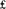
*p* = 0.000; week four compared to week two,

‡ *p* = 0.000.

### Osteophyte Formation

The tibial plateau was the site of significant osteophyte formation, which was found on the medial and lateral sides in all rabbits as early as week four post-surgery. The mean size of osteophytes markedly increased over time (*p* = 0.000; Table 3).

**Table 3:** Osteophyte Size Measured by Magnetic Resonance Imaging (MRI) & Macroscopic Examination.

		Media tibial lateau (mm)	Lateral tibial lateau (mm)	Total (mm)
**Baseline**	Osteophyte size by MRI	0.00	0.00	0.00
	Osteophyte size by macroscopic examination	0.00	0.00	0.00
**Week two**	Osteophyte size by MRI	1.06±0.34	0.87±0.33	0.97±0.35
	Osteophyte size by macroscopic examination	1.43±0.32	1.19±0.32	1.31±0.34
**Week four**	Osteophyte size by MRI	1.87±0.30*	1.24±0.38▴	1.55±0.47 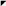
	Osteophyte size by macroscopic examination	2.34±0.52•	1.67±0.37⊕	2.00±0.56▽
**Week eight**	Osteophyte size by MRI	3.21±0.51^§^	2.21±0.47¶	2.71±0.70★
	Osteophyte size by macroscopic examination	3.73±0.43#	2.82±0.45 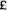	3.27±0.63▾

Data (n = 20 knees) are expressed as means±SDs.

Osteophyte size measured by MRI in media tibial plateaus, week eight compared to weeks four and two, §*p* = 0.000; week four compared to week two,

*p = 0.000. Osteophyte size measured by macroscopic examination in medial tibial plateaus, week eight compared to weeks four and two,

# *p* = 0.000; week four compared to week two,

• *p* = 0.000.

Osteophyte size measured by MRI in lateral tibial plateaus, week eight compared to weeks four and two,

¶ *p* = 0.000; week four compared to week two,

▴ *p* = 0.000. Osteophyte size measured by macroscopic examination in lateral tibial plateaus, week eight compared to weeks four and two,

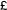
*p* = 0.000; week four compared to week two,

⊕ *p* = 0.000.

Osteophyte size measured by MRI in total tibial plateaus, week eight compared to weeks four and two,

★ *p* = 0.000; week four compared to week two,

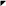
*p* = 0.000. Osteophyte size measured by macroscopic examination in total tibial plateaus, week eight compared to weeks four and two,

▾ *p* = 0.000; week four compared to week two,

▽ *p* = 0.000.

### Subchondral Bone Edema

Subchondral bone edema – as indicated by a MRI hypersignal – was frequently observed as early as week two post-surgery on the medial femoral condyles and tibial plateaus. The score and grade significantly increased with time (*p*<0.05; Table 4). The lesions were primarily seen in the posterior section of the tibial plateaus and femoral condyles. Over time, they were also found on the lateral regions of the femoral condyles, the tibial plateaus, and the trochlea (Fig. 1); however, the score and grade were more pronounced in the medial compartment than those in the lateral compartment.

**Table 4:** Subchondral Bone Lesion Scores and Grades Measured by Magnetic Resonance Imaging (MRI).

		Femoral condyles nd trochlea	Tibial plateaus	Medial ompartment	Lateral ompartment
**Baseline**	Score	0.00	0.00	0.00	0.00
	Grade	0.00	0.00	0.00	0.00
**Week two**	Score	15.67±1.53	17.00±1.41	17.50±0.71	15.00±1.41
		(17:14:16)	(18:16)	(17:18)	(14:16)
	Grade	0.78±0.42	0.85±0.36	0.90±0.33	0.88±0.30
**Week four**	Score	25.33±2.08*	31.50±2.12▴	30.00±4.24▾	26.5±4.95†
		(20:20:20)	(20:20)	(20:20)	(20:20)
	Grade	1.27±0.45•	1.58±0.64 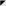	1.50±0.64Ф	1.33±0.47‡
**Week eight**	Score	40.33±2.52§	43.00±4.24¶ ^★^	44.50±2.12▪	39.00±1.41 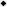
		(20:20:20)	(20: 20)	(20: 20)	(20: 20)
	Grade	2.01±0.65#	2.15±0.74 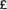	2.23±0.53 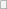	1.95±0.60 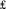

Data (n = 20 knees) are expressed as means±SDs. Scores are expressed as the summation of grades, and the grades as an average of the grades obtained for each region. Incidences of the lesions in subregions are indicated in parentheses (number of rabbits with lesions on medial/lateral femoral condyles/trochlea, medial/lateral tibial plateaus, or femoral condyles/tibial plateaus in the compartments).

Femoral condyles and trochlea score: week eight compared to weeks four and two,

§ *p* = 0.000; week four compared to week two,

^*^ *p* = 0.001. Femoral condyles and trochlea grade: week eight compared to weeks four and two,

# *p* = 0.000; week four compared to week two,

• *p* = 0.000.

Tibial plateaus score: week eight compared to week four,

¶ *p* = 0.028; week four compared to week two,

▴ *p* = 0.015; week eight compared to week two,

★ *p* = 0.003. Tibial plateaus grade: week eight compared to weeks four and two,

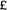
*p* = 0.000; week four compared to week two,

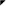
*p* = 0.000.

Medial compartment score: week eight compared to weeks four and two,

▪ *p* = 0.000; week four compared to week two,

▾ *p* = 0.018. Medial compartment grade: week eight compared to weeks four and two,

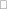
*p* = 0.000; week four compared to week two,

^Φ^ *p* = 0.002.

Lateral compartment score: week eight compared to weeks four and two,

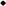
*p* = 0.000; week four compared to week two,

† *p* = 0.002. Lateral compartment grade: week eight compared to weeks four and two,

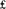
*p* = 0.000; week four compared to week two,

‡ *p* = 0.002.

### Interobserver Agreement and Correlations between MRI and Macroscopic Assessment

**Figure 2:**
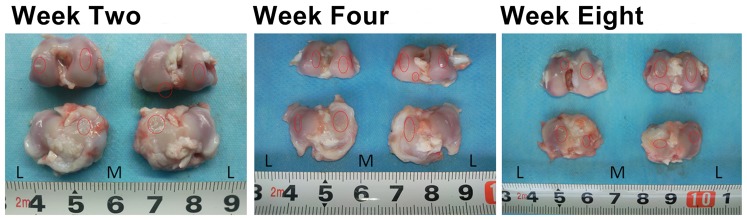
Macroscopic Appearance of Osteoarthritic Cartilage from Weeks Two through Eight Post-Surgery. L, lateral; M, medial. Circles indicate lesion areas (mm).

**Figure 3:**
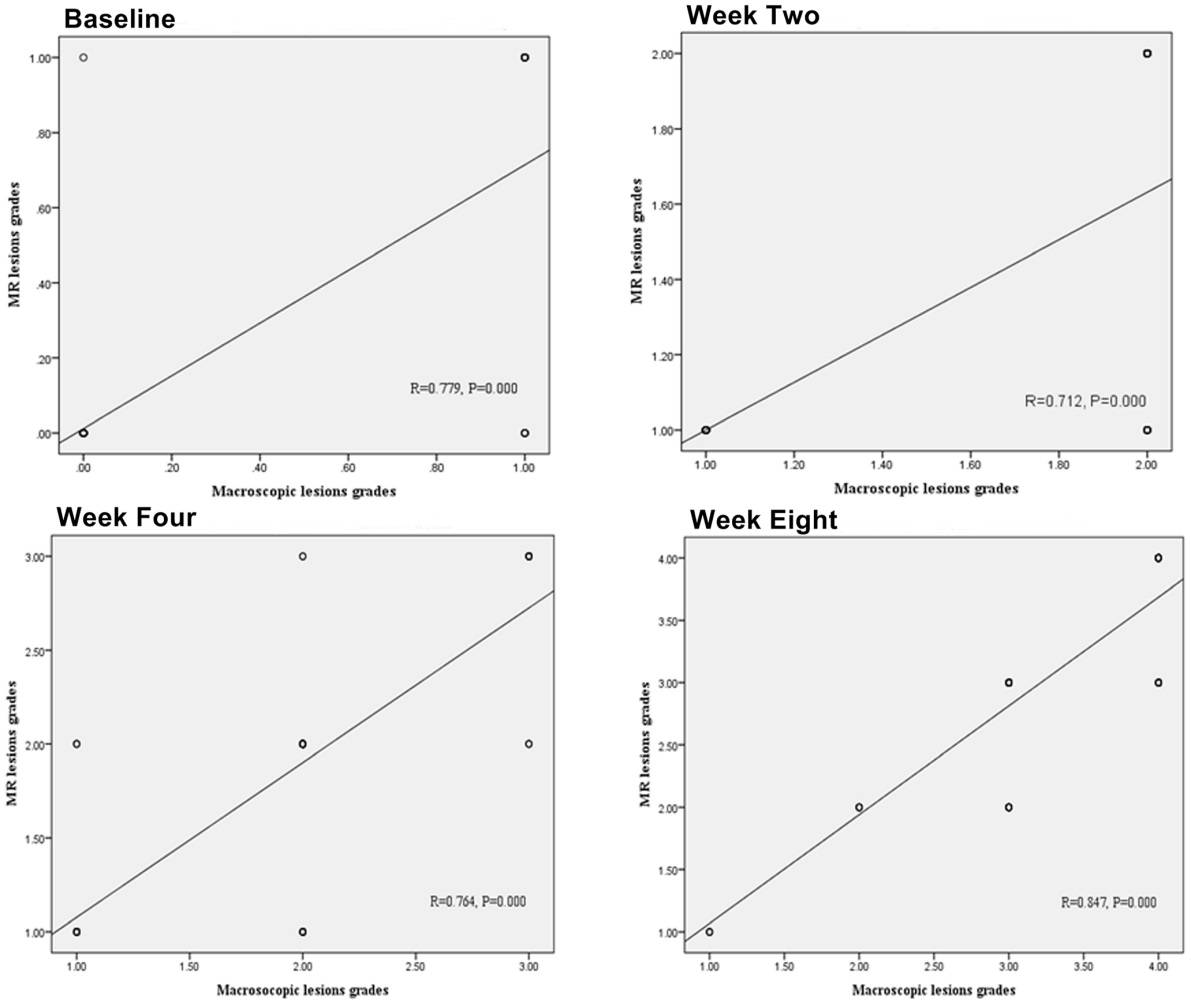
Correlations between Magnetic Resonance Imaging and Macroscopic Lesion Grading at Different Time Points (n = 20 knees).

The weighted kappa statistics for interobserver agreements for MRI cartilage defect grading between the two radiologists were 1.000, 0.824, 0.835, and 0.841 for each successive time point, respectively (*p* = 0.000), while the weighted kappa statistics for interobserver agreements for macroscopic cartilage defect grading between the two orthopedic surgeons were 1.000, 0.861, 0.822, and 0.822 for each successive time point, respectively (*p* = 0.000). The locations and grading of cartilage defects by macroscopic evaluation were found to be in agreement with those found by MRI (Fig. 2). Statistically significant correlations were found between MRI and macroscopic lesion grades at every time point (*p* = 0.000) (Fig. 3).

## Discussion

The construction of a model of surgically-induced osteoarthritis in rabbits was initially reported by Hulth and his colleagues in the 1970's. In their rabbit model, the knee joints displayed no pathological changes until day 15 post-surgery at which time the first degenerative changes appeared. After day 30, the clusters were more commonly observed along with some flaking of the superficial layer [6]. Consistent with this early study, we also observed changes in chondral and subchondral structures at weeks two, four, and eight post-surgery. The present study provides novel insight into the evolution of changes in knee chondral and subchondral structures in this surgically-induced OA rabbit model as assessed by MRI and demonstrates that the progressive alterations in chondral and subchondral structures can be accurately followed and semi-quantified by MRI.

In both epidemiological studies and clinical trials, OA progression has been conventionally assessed by estimating cartilage loss through measuring the narrowing of joint spaces via radiographic imaging [25]–[27]. Although this method is the current gold standard for OA, this radiographic method is of limited value in imaging articular cartilage, since this technique can only indirectly assess changes in cartilagenous tissue [28]. In contrast, MRI – by virtue of its superior soft-tissue contrast, lack of ionizing radiation, and multiplanar capabilities – is superior for the evaluation of articular cartilage [29], [30]. Moreover, X-ray radiography and MRI differ in that the former is performed under weight-bearing conditions while the latter is not. Thus, MRI is especially useful for imaging of an animal model of OA under non-weight-bearing conditions.

Several recent publications have described the use of fat suppressed three-dimensional spoil gradient-recalled sequences for the evaluation of knee hyaline cartilage, which has shown greater sensitivity and specificity in detecting hyaline cartilaginous defects [29], [31]–[35]. However, these sequences generally require long acquisition times and additional time for off-line manipulation to create images. Animals can produce motion artifacts during long acquisition periods that adversely affect MRI quality. To address this issue here, we used the T2-FI3D-we-sag sequence that requires a shorter acquisition time (3 min 25 s) compared to conventional MRI sequences (25 min or longer) [36]. Therefore, this T2-FI3D-we-sag sequence-based MRI method should be superior to conventional MRI techniques for detecting articular cartilaginous abnormalities in animal models of OA and requires further study.

The assessment of structural changes by MRI had been previously investigated in both the ACLT canine model of OA [21] and guinea pig spontaneous model of OA [37]. However, the changes in chondral and subchondral structures in this rabbit model have not been thoroughly investigated yet. The canine study only transected the anterior cruciate ligament (ACLT) [21] and joint disease progresses at a faster rate in rabbits [38], which may account for the slower progression of joint changes in the canine study as compared to the current study. Thus, based on the rabbit's smaller size, lower cost, and faster rate of disease progression, this rabbit OA model should significantly shorten the cost and time frame for future DMOADs studies.

Cartilagenous changes were detected earliest in the disease process. The weighted kappa statistic for interobserver agreement between the two radiologists assessing the cartilaginous changes were 1.000, 0.824, 0.835, and 0.841 at each time point, respectively, indicating almost perfect agreement [24]. Cartilaginous changes were found in almost all subregions at week two post-surgery and progressed from a grade of 1.15–1.30 (indicating the presence of cartilagenous edema and minimal loss of cartilage) to a grade of 3.00–3.35 by week eight post-surgery (indicating the presence of severe cartilagenous erosion). These findings were corroborated by macroscopic examination at weeks two, four, and eight post-surgery. The progressive increase in the scores and grades of early lesions in the subregions is suggestive of the worsening of existing lesions as opposed to the development of novel lesions over time. The detection of cartilagenous edema by MRI in early lesions is in line with Calvo et al.'s [36] rabbit meniscectomy model and Boileau et al.'s [21] canine ACL model. The severity and prevalence of cartilagenous defects of the knee have been significantly associated with osteophytes and bone size [39]. The present study also demonstrated a predominance of cartilagenous lesions on the tibial plateaus over the femoral condyles and trochlea, which is in line with previous human studies showing a preferential development of cartilagenous lesions on the weight-bearing areas of the femoral condyles and tibial plateaus with a predominance of cartilagenous loss in the medial compartment [40]–[43].

Subchondral bone edema has long been recognised as important in terms of the pain and progression of OA [44], and an increase in the size of subchondral bone edematous lesions has been found to be correlated with the loss of cartilage over time in OA patients [45]. Moreover, subchondral bone edema in the ACLT canine model [21] has been found to correspond to the area of bone marrow necrosis, fibrosis, and abnormal trabecular remodelling with these structural changes being more pronounced at the location of the cartilagenous lesions (the medial compartment), suggesting a close relationship between cartilagenous lesions and subchondral bone edema. Here, subchondral bone edema appeared at week two post-surgery, and we observed a progressive increase in the size and number of subregions with subchondral bone edema over time. Specifically, subchondral bone lesions progressed from a grade of 0.00 (baseline) to 0.78–0.90 (week two) to 1.27–1.58 (week four) to 1.95–2.23 (week eight) post-surgery. However, the positive rate of subchondral bone edema was lower than that of cartilagenous changes at week two post-surgery; therefore, it can be deduced that subchondral bone edema occurs secondary to cartilagenous changes in this OA model. Notably, both cartilagenous defects and subchondral bone edema scores and grades were more pronounced in the medial compartment. Rabbits, unlike humans, more predominantly bear weight on the lateral compartment of the knee; thus, the fact that damage was predominantly found in the medial compartment is as much of a reflection of the surgical technique as an alteration in normal joint loading.

Osteophytes are a classic OA classification and have been strongly associated with radiographic joint space narrowing, subchondral sclerosis, and pain [46]–[48]. Recent studies have demonstrated that osteophytes are associated with cartilage defects in the tibiofemoral and patello-femoral joints [49]. In current study, the size of osteophytes on the tibial plateau were greater than those on the femoral condyles or trochlear ridges, and the size of osteophytes on the medial tibial plateau were greater than those on the lateral tibial plateau. Addtionally, the osteophyte size evaluated by MRI at weeks two, four, and eight was slightly smaller than those evaluated by visual assessment, suggesting that the cartilage covering the bone structure could not be visualized on the MRI sequence used to measure the osteophytes [21] and demonstrates that MRI may not accurately detect osteophyte size.

In this study, cartilage defects were found in almost all subregions at week two and progressed from a grade of 1.15–1.30 at week two to 1.90–1.97 at week four (indicating chondral softening or edema with an intact surface [23]) to severe lesions with a grade of 3.00–3.35 at week eight (indicating the presence of severe erosion). According to the results of a previous study [29], grades zero and one are considered OA-negative, while grades two, three, and four are considered OA-positive; on this basis, the current model was OA-negative at week four post-surgery and OA-positive at week eight post-surgery. Additionally, cartilage volume correlates well with MRI grading of articular cartilage [50]. The cartilagenous edema observed at week four post-surgery indicates that the cartilagenous lesions measured at this timepoint may have been underestimated in the previous study [36]. Therefore, in order to evaluate the effectiveness of new DMOADs at an early OA stage, this animal model should be used prior to week four post-surgery; otherwise, this model should be used at week eight post-surgery. In addition, a significant correlation between MRI and macroscopic appearance allows researchers to assess joint appearance without sacrificing the rabbit, which should facilitate the development of superior research protocols for this animal model.

There are several limitations to this study. First, this surgically-induced OA model cannot duplicate the natural course of OA, as OA's structural changes take place over decades in humans; therefore, alternative animal models of OA, such as the spontaneous OA model, may be more suitable for future studies. Second, the current MRI results should be compared with computed tomography and radiography to illustrate their strengths and weaknesses for future clinical investigations. Third, quantitative measurement of cartilage defect volume and surface area should be performed in future studies on this animal model. Fourth, as the extent of animal activity was not quantified here, we were unable to correlate animal activity levels with MRI and macroscopic grading; thus, further research with respect to this question is required.

## Conclusions

This study provides valuable insights on the usefulness of MRI in assessing the evolution of changes in osteophytic, chondral, and subchondral structures in a surgically-induced OA rabbit model. This study also provides information about the progressive nature of the osteophytic, chondral and subchondral structural changes in this animal model as well as its similarities and differences to the course of OA in humans. Specifically, week four post-surgery was considered the timepoint between OA-negative and OA-positive status in this animal model. The combination of this OA model with MRI evaluation should provide a promising tool for the pre-clinical evaluation of new DMOADs.

## Supporting Information

Data S1 (DOCX)
